# Supplementary material for: Multiplex-GAM: genome-wide identification of chromatin contacts yields insights overlooked by Hi-C
Source: Nat Methods. 2023 Jun 19;20(7):1037–47. doi: 10.1038/s41592-023-01903-1 (PMC10333126; doi:10.1038/s41592-023-01903-1)
Supplement: Supplementary file 1 — Supplementary Table 1, legends for Supplementary Tables 2–10, and Supplementary Notes for SLICE. [file 41592_2023_1903_MOESM1_ESM.pdf]

nature methods

Article https://doi.org/10.1038/s41592-023-01903-1

Multiplex-GAM: genome-wide identification of chromatin contacts yields insights overlooked by Hi-C

In the format provided by the authors and unedited

Legends for Supplementary Tables 2-10

Supplementary Table 2	Summary of **GAM samples**
Supplementary Table 3	Parameter **estimates** obtained through **SLICE** modelling
Supplementary Table 4	List of pairwise **SLICE FDR contacts**
Supplementary Table 5	Summary of **published feature sets**
Supplementary Table 6	**Feature** sets **used in Random Forest** analysis
Supplementary Table 7	**Summary of specific contacts** obtained using 5% and 10% most differential contacts
Supplementary Table 8	**Feature pair enrichments** found in the 5% contact sets
Supplementary Table 9	**Feature pair enrichments** found in the 10% contact sets
Supplementary Table 10	**Slices intersecting nuclei**

Supplementary Table 1

	Bulk Hi-C	Single-cell Hi-C	GAM
Input cells	Typically >100,000 cells	Typically 500-1000 cells	Typically 500-1000 cells
Measurement of complex, multi-partner interactions	Underestimates pairwise interactions for complex loci (due to ligation)	Underestimates pairwise interactions for complex loci (due to ligation)	No ligation required, therefore accurately measures interactions of complex loci
Preparation of cells	Requires tissue dissociation and purification of target cell type, which can alter cell physiology (van den Brink et al., 2017), compromise cell identification (Millard et al., 2021) and cause differential loss of cell types (Denisenko et al., 2020; especially in diseased tissue, Korin et al., 2021)	Requires tissue dissociation and purification of target cell type	Tissue is dissected intact and fixed without cell dissociation Target cells directly extracted by laser microdissection (Winick-Ng et al., 2021)[30]
Tissue geography	Does not preserve locations of targeted cells within tissue	Does not preserve locations of targeted cells within tissue	Recording location of cells within tissue is possible
Radial positioning	Does not measure radial positioning	Radial position can be extracted by 3D modelling	Measures radial positions (Beagrie et al., 2017)[3]
Measurement of distances	Accurately captures average distances between loci (Fiorillo et al., 2021)[9]	Individual cells poorly capture distances between loci (Fiorillo et al., 2021)[9]	Accurately captures average distances between loci with better signal-to-noise ratio than Hi-C at distances >1Mb (Fiorillo et al., 2021)[9]
Fixation protocol	Mild or no fixation with risk of chromatin collapse	Mild fixation with risk of chromatin collapse	Strong fixation: preservation of nuclear ultrastructure validated by Electron Microscopy (Guillot et al., 2004)[20]

Additional references:

Denisenko, E., Guo, B.B., Jones, M. *et al.* Systematic assessment of tissue dissociation and storage biases in single-cell and single-nucleus RNA-seq workflows. *Genome Biol* **21**, 130 (2020). https://doi.org/10.1186/s13059-020-02048-6

Korin, B., Chung, J.J., Avraham, S., Shaw, A.S. Preparation of single-cell suspensions of mouse glomeruli for high-throughput analysis. *Nat Protoc*. **16**, 4068-4083 (2021). https://doi.org/10.1038/s41596-021-00578-2

Millard, S.M., Heng, O., Opperman, K.S. *et al.* Fragmentation of tissue-resident macrophages during isolation confounds analysis of single-cell preparations from mouse hematopoietic tissues. *Cell Reports* **37**, 110058 (2021). https://doi.org/10.1016/j.celrep.2021.110058

van den Brink, S., Sage, F., Vértesy, Á. *et al.* Single-cell sequencing reveals dissociation-induced gene expression in tissue subpopulations. *Nat Methods* **14**, 935–936 (2017). https://doi.org/10.1038/nmeth.4437

Supplementary Notes

In this Supplementary Note, we will discuss the mathematical implementation of the SLICE (StatisticaL Inference of Co-sEgregation) model and its new features. SLICE allows the quantification of the interaction frequencies between specific DNA loci from GAM (Genome Architecture Mapping) raw data, by correcting for both technical (e.g., detection efficiency) and biological (e.g., genomic distance) factors (Beagrie et al., 2017).

In **Section 1**, we briefly summarize the general mathematical framework of SLICE. **Section 2** is devoted to the extensions of the original SLICE implementation (Beagrie et al., 2017) to model nuclear shape (**Section 2.1**), cellular ploidy (**Section 2.2**) and the multiplex-GAM experimental setup (**Section 2.3**). Finally, we describe how SLICE can be employed to optimize GAM experimental protocols (**Section 3**) and to combine different GAM datasets (**Section 4**).

1. SLICE model

A GAM experiment consists of the collection of many nuclear cryosections (nuclear profiles or NPs) in random orientations from a population of nuclei, with one NP being isolated from each nucleus. The DNA content of each NP is then sequenced to provide information about presence/absence of any DNA locus. SLICE is a statistical model that, starting from raw GAM data, estimates the interaction probability between two or more chromatin loci.

In the following, we briefly introduce the SLICE model focusing first on the case of pairwise (**Section 1.1**) and threewise (**Section 1.2**) interactions. For additional details, we refer the reader to Beagrie et al., 2017.

1.1 Pairwise interactions

In the SLICE model, we assume that a pair of loci, *A* and *B*, can be in two different states: in an interacting state, when the two loci are spatially close even if genomically distant, or in a non-interacting state, when they are separated from each other with a physical nuclear distance that is dependent on their genomic separation. These states occur with probabilities *Pi* and *(1-Pi)* respectively. In addition, we define state-specific probabilities for the loci *A* and *B* to be segregated in a randomly selected slice (Suppl. Notes Figure SN1) as follows:

- v_1 and v_0 are the probabilities to find/not find a single locus in the slice;
- u_2, u_1, u_0 are the probabilities to find 2, 1 or 0 loci in a pair of non-interacting loci;
- t_2, t_1, t_0 are the probabilities to find 2, 1 or 0 loci in a pair of interacting loci.

The following normalization relationships are satisfied:

- $u_2 + 2u_1 + u_0 = 1$
- $t_2 + 2t_1 + t_0 = 1$
- $v_1 + v_0 = 1$

These probabilities depend on the geometric properties of the system. For instance, in the case of spherical nuclei, the probabilities v_0 and v_1 can be analytically calculated and depend on the ratio between the slice thickness and the nuclear radius (Beagrie et al., 2017). Analogously, the

probabilities for pairs of loci (i.e. t_0, t_1, t_2 and u_0, u_1, u_2) depend also on the average physical distance between the loci in the interacting and non-interacting states, and can be estimated numerically (e.g., via Monte Carlo simulations) or from GAM data (Beagrie et al., 2017). However, for loci at large genomic distances (≳50Mb) and small physical distances d_I when interacting ($d_I \lesssim h$), the following approximations can be used (see Beagrie et al., 2017):

$$u_0 v_0^2, u_1 v_1 v_0, u_2 v_1^2$$
$$t_0 v_0, t_1 0, t_2 v_1$$

From these probabilities, SLICE can predict the outcome of a GAM experiment as a function of the interaction probability *Pi* of a pair of loci. For example, if only one nuclear profile is added per tube, the fraction of tubes that include neither *A* nor *B* loci in a diploid nucleus is:

$$M_0 = N_{0,0} = [Pi\ t_0 + (1-Pi)u_0]^2$$

where $N_{0,0}$ is the fraction of slices with neither *A* nor *B* loci.
Similarly, we can calculate the fraction of tubes including two (M_2) or one (M_1) loci (Beagrie et al., 2017). Conversely, *Pi* can then be estimated by comparing the predictions of M_2, M_1, M_0 with the experimental values measured by GAM (Beagrie et al., 2017).

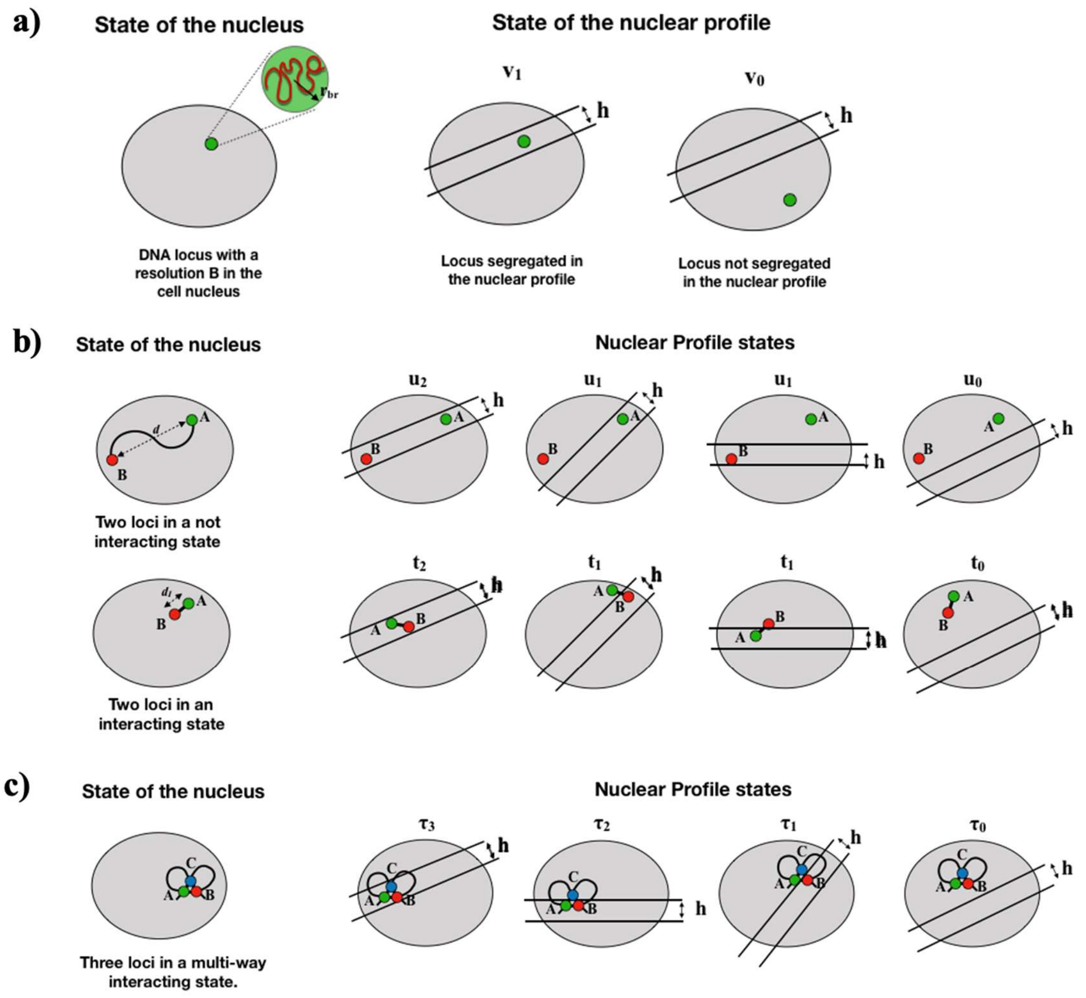

Figure SN1: SLICE model parameters.

a) Schematic picture of a DNA locus in the cell nucleus with a given resolution b_r. Each locus is represented by a sphere with a radius r_{b_r}, that depends on the considered resolution b_r. The probabilities to find or not a locus in a population nuclear profiles, with slice thickness h, are v_1 and v_0 respectively.

b) The probability to find a pair of loci in a population of nuclear profiles depends on whether they are in a not-interacting (u_i, top) or interacting (t_i, bottom) state.

c) Probability τ_i of finding 3, 2, 1 or 0 loci of a triplet in a interacting state.

1.2 Threewise (triplet) interactions

SLICE can exploit the co-segregation information from GAM to estimate the frequency of multivalent interactions between DNA loci. For example, the interaction probability of triplets of loci, Pi_3, can be estimated following an approach similar to the pair-wise calculation. Briefly, SLICE can estimate how often any combination of the loci in the triplet are co-segregated in the same nuclear slice in the case when the three loci are interacting or not interacting.

As an example, we consider the case where all the three loci *A, B* and *C* are interacting and define τ_i the probability that i (i = 0, 1, 2, 3) loci of the triplet are co-segregated in the same

nuclear profile. Again, these probabilities can be numerically estimated. However, if the physical distances of the loci interacting in the triplet is $\lesssim h$, h being the slice thickness, the probabilities τ_i are well approximated by:

$$\tau_0 \sim v_0,\ \ \tau_1 \sim \tau_2 \sim 0,\ \tau_3 \sim v_1$$

where v_1 and v_0 have been previously defined.
Analogously, SLICE can calculate the probabilities corresponding to the cases where only two loci of the triplet are interacting or the three loci are not interacting (Beagrie et al, 2017). These quantities can be used to derive the equations for the fraction of nuclear profiles $N_{i,j,k}$ (with i, j, k = 0, 1) containing any combination of the loci. Hence, the fraction of tubes containing any number of loci, which are the quantities measured by GAM and can be fitted to determine the triplet interaction probability (Beagrie et al., 2017).
In those calculations, additional complexities can be taken into account, such as the detection efficiency of loci in a tube or the effects of genomic resolution (as discussed in Beagrie et al, 2017).

2. SLICE model extensions

In its previous implementation, SLICE considered diploid cells with nuclei approximated as spheres (Beagrie et al., 2017). Here, we show how the model can be generalized to include different nuclear shapes (**Section 2.1**) and ploidies (**Section 2.2**). Finally, in **Section 2.3** we extend SLICE to model the new multiplex-GAM protocol, which reduces hands-on time and costs.

2.1 Modelling the nuclear shape

SLICE can model an arbitrary nuclear shape by extending the computation of the probabilities v_i, u_i and t_i defined above. In general, these can be numerically estimated (e.g., via Monte Carlo methods). For the sake of simplicity, we consider ellipsoids, which are good approximations of nuclear shapes for a number of cell types (**Supplementary Tables 3**) and can be treated analytically.
Below, we calculate the analytical expression of v_1 in ellipsoidal nuclei, which allows the estimation of the probabilities u_i and t_i for pairs of loci at large genomic distances ($\gtrsim$50Mb) and small physical distances d_I when interacting ($d_I \lesssim h$; see above); for all other cases, numerical simulations or GAM data can be used, as described in (Beagrie et al, 2017). The results can be easily generalized to take into account different experimental detection efficiencies or resolutions (Beagrie et al., 2017).

2.1.1Segregation probability v_1 for ellipsoidal nuclei

We consider a cell nucleus with an ellipsoidal shape and semi-axes *a, b, c*. Without loss of generality, any ellipsoid can be described by the canonical implicit equation:

$$\frac{x_1{}^2}{a^2}+\frac{x_2{}^2}{b^2}+\frac{x_3{}^2}{c^2}=1 \qquad \text{(Eq. 1)}$$

where x_1, x_2 and x_3 are the 3D coordinates.
In a coordinate system defined by the following anisotropic scaling transformation:

$$(x_1, x_2, x_3) \rightarrow \left(x'_1=\frac{x_1}{a}, x'_2=\frac{x_2}{b}, x'_3=\frac{x_3}{c}\right) \qquad \text{(Eq. 2)}$$

the ellipsoid is transformed into a sphere with unit radius. Hence, in this new coordinate system (indicated by apex symbols) we can use the equations for v_1 and v_0 written in the spherical case (see Beagrie et al., 2017), with a slice thickness h' defined by:

$$h' = \frac{h}{\sqrt{(a sin cos)^2 + (b sin sin)^2 + (c cos)^2}} \qquad \text{(Eq. 3)}$$

where θ and ϕ are respectively the azimuthal and polar angle identified by the direction $\hat{n}$ of the slice plane (**Suppl. Notes Figure SN2a**). The average segregation probability for a single locus, v_1', can be obtained by integrating over all possible slice orientations and positions, as follows:

$$v_1' = \frac{\int_S d\Omega \int_{-h'(,)/2}^{1} dz'\, v_1'(z',\hat{n})}{\int_S d\Omega \int_{-h'(,)/2}^{1} dz'} \qquad \text{(Eq. 4)}$$

where $\int_S d\Omega \; = \int_0^{2} d\theta \int_0 \; d \sin$ is the integral over the solid angle, $v_1'(z,\hat{n})$ is the segregation probability of a single locus in a slice of a sphere with orientation $\hat{n}$ and distance from the equatorial plane equal to z. Since the transformation (**Eq. 2**) is linear, the associated Jacobian determinant is constant. Therefore the volumes ratios are invariant, and then v_1' is equal to v_1.
As an example, in **Suppl. Notes Figure SN2b** we show the value of v_1 computed by numerical integration of equation (**Eq. 4**) for different values of semi-axis lengths (a, b, c), as a function of the slice thickness *h*. The values computed from **Eq. 4** match those obtained by Monte Carlo simulations (marked by circles).
Given the above equation for v_1, we can estimate the probabilities u_i and t_i and the fraction of tubes with 2, 1 or 0 loci (M_2, M_1, M_0) in the case of an ellipsoidal nucleus, with the calculations mentioned in **Section 1** and explained in detail in Beagrie et al., 2017.

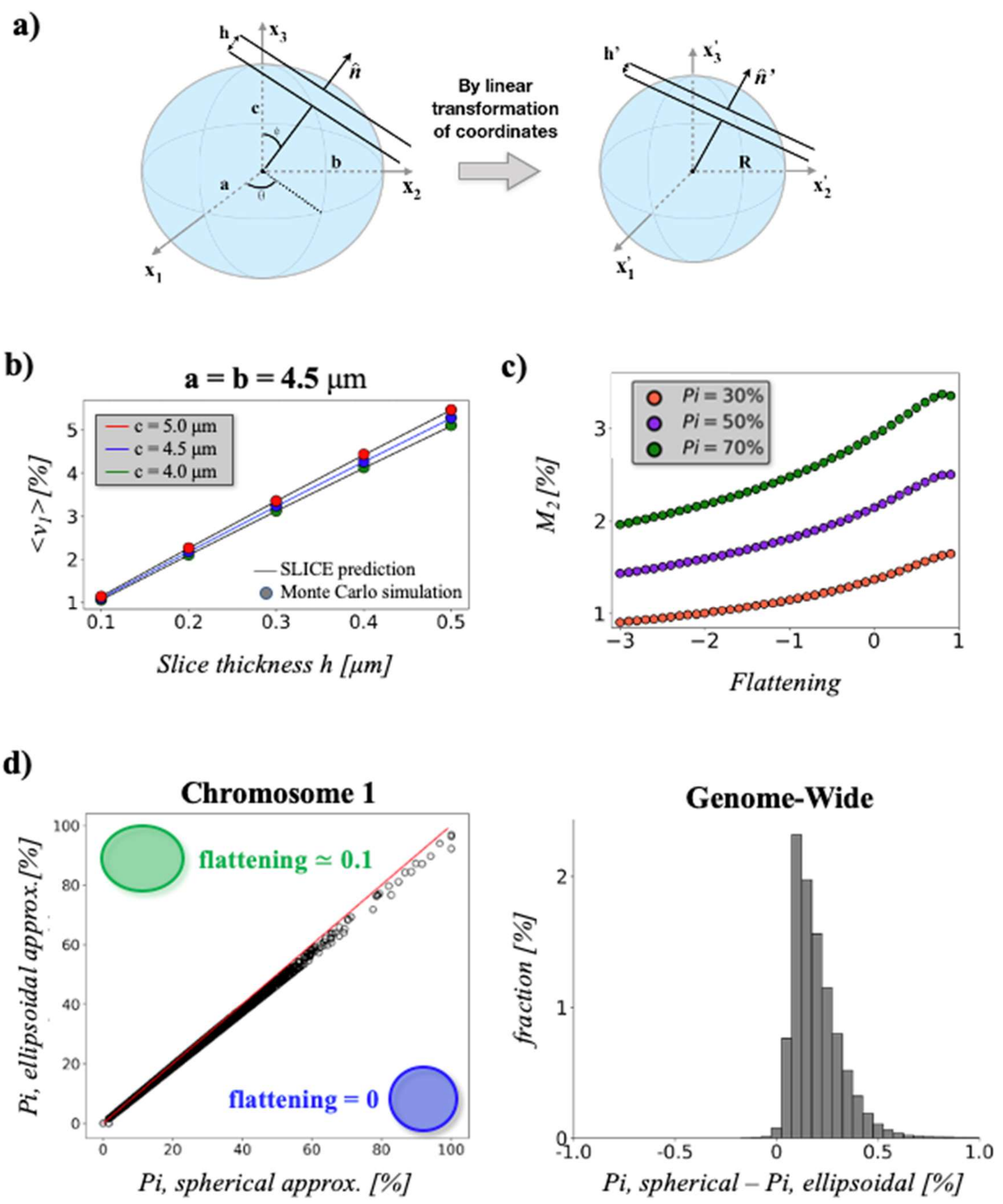

Figure SN2: SLICE generalization to ellipsoidal nuclei.

a) Schematic picture showing the linear transformation of coordinates, from (x_1, x_2, x_3) to (x_1', x_2', x_3'), for mapping a generic ellipsoid with semi-axis length (a, b, c) to spherical case, with unitary radius (*R*=1).

b) Average probability v_1 that a locus is found in a nuclear profile from a population of ellipsoidal nuclei, as a function of the slice thickness *h* and for different lengths of semi-axes. Here, we show the spherical case (a = b = c = 4.5μm) and the cases where we increase/decrease the semi-axis length by about 10%. As expected, we find that v_1 decreases (increases) if we consider a smaller (bigger) nuclear volume. The SLICE prediction (solid lines) matches with independent Monte Carlo simulations (circles).

c) Co-segregation frequency M_2 as a function of the nucleus *flattening* parameter, for different values of interaction probability *Pi*. Unlike **Extended Data Fig. 3d**, here we considered variations in semi-axis lengths leading to changes of the ellipsoid volume *V*. We fixed slice thickness h=*220 nm*, b_r=*30 kb* and detection efficiency $\varepsilon=1$ and we considered two loci on different chromosomes, and therefore randomly distributed within the nucleus.

d) Comparison of SLICE results on multiplex-GAM data at 40 *kb* resolution, in spherical (*R*=4.5 μm) and ellipsoidal approximation, where we increased the length of one semi-axis of about 10% (a = b = 4.5, c = 5.0 μm; *flattening*≃0.1). (Left) Scatterplot of interaction frequencies *Pi* for chromosome 1 in spherical and ellipsoidal approximation. (Right) Distribution of differences in *Pi* values genome-wide between spherical and elliptical case. For sake of simplicity we only show differences between non-zero *Pi* values in both cases.

2.1.2 Finite genomic resolution of loci

In **Eq.4,** we assumed that the genomic region we want to detect is a dimensionless point. However, to take into account for the finite size of a locus at a given genomic resolution, we can represent a DNA locus including b_r bases as a sphere with a finite radius r_{b_r} (**Suppl. Notes Figure SN1a**). It can be shown that, if r_{b_r} << a, b, c (i.e., the semi-axes of the ellipsoidal nucleus), the previous equations still hold if *h* is replaced by an effective slice thickness $h_{eff} = h + 2r_{b_r}$ (Beagrie et al., 2017). An estimation of r_{b_r} can be obtained under the assumption that the genetic material is uniformly distributed within the cell nucleus, which leads to:

$$r_{b_r} = \left(\frac{b_r}{L}\ \mathrm{abc}\right)^{1/3} \qquad \text{(Eq. 5)}$$

where *L* is the total length of the genome (Beagrie et al., 2017).

2.1.3 Effects of nuclear shapes

In this section, we use the equations for ellipsoidal nuclei to investigate how the nuclear shape can affect the co-segregation frequency M_2, i.e. the expected fraction of tubes containing both loci of a pair (**Section 1**). Moreover, we test how robust are the SLICE calculations obtained in mES cells to changes of the nuclear shapes.

While the above equations hold true for an arbitrary ellipsoid, here, for the sake of simplicity, we focus on spheroids, i.e., ellipsoids with two equal semi-axes (a = b) (**Extended Data Fig. 3a**). In this way. we can parametrize the nuclear shape by using the *flattening*, defined as 1 - c/a. By varying the length of the c-axis, we can consider two possible cases: the a and b semi-axes are kept constant, leading therefore to a variation of the total volume, or, alternatively, the volume is kept constant, and the two semi-axes a and b change accordingly. For both cases. we derived the co-segregation frequency of pairs of loci (M_2) as a function of the *flattening* parameter (shown in **Extended Data Fig. 3b** and **Suppl. Notes Figure SN2c**

respectively). In general, we found that M_2 changes non-trivially with *flattening* and exhibits a different behaviour with respect to volume variation, proving that not only the nucleus shape, but also its size affects the co-segregation frequency.
Next, we quantified how robust are our SLICE results obtained under the assumption of perfectly spherical nucleus. To this aim, we compared the results obtained on combined Multiplex-GAM data at 40 kb resolution in spherical approximation against the results obtained by introducing a small flattening ($a = b = R = 4.5\ \mu m$, $c = 5\ \mu m$, *flattening* ≈ 0.1). In both cases, SLICE finds very similar results (**Suppl. Notes Figure SN2d**). In particular, this change in the shape leads to a slight shift of interaction probability *Pi*, as quantified by Pearson correlation coefficients (values above the 99%) and overlaps of contacts (about 98% for all the chromosomes). Additionally, the estimated detection efficiencies ε are very similar (88% ellipsoidal case against 86% in spherical case).

2.2Modelling cellular ploidy and detection efficiency

To extend SLICE to cells with any chromosome copy number (ploidy), we generalized the equations for the $N_{i,j}$, i.e., the expected fraction of nuclear profiles including *i* copies of locus *A* and *j* copies of locus *B* (see above) in a population of *N* cells. In the following, for the sake of simplicity, we focus on the cases of haploid, diploid and triploid cells, but SLICE equations can be easily generalized to arbitrary ploidies.
For diploid cells, where *i* and *j* assume values between 0 and 2, $N_{i,j}$ can be written in mean field approximation as a function of $c_0 = Pi \cdot t_0 + (1-Pi) \cdot u_0$ and v_0, i.e., the probabilities that a pair of loci or a single locus are not contained in a nuclear profile, respectively (Beagrie et al., 2017). More specifically, the $N_{i,j}$ are polynomial functions of v_0 and c_0 with a degree equal to the ploidy of the cells.
For example, in haploid cells the expressions for $N_{i,j}$ are linear combinations of v_0 and c_0:

$$\begin{aligned} N_{0,0} &= c_0 \\ N_{1,0} = N_{0,1} &= (v_0 - c_0) \qquad \text{(Eq. 6)} \\ N_{1,1} &= (1 - 2v_0 + c_0) \end{aligned}$$

On the other hand, for triploid cells the $N_{i,j}$ are third degree polynomial functions in v_0 and c_0. For instance, $N_{1,1}$, i.e., the fraction of nuclear profiles including one locus *A* and one locus *B* in a triploid cell is:

$$N_{1,1} = 3\,(1 - 2v_0 + c_0)\,c_0^2 + 6\,(v_0 - c_0)^2 c_0 \qquad \text{(Eq. 7)}$$

SLICE can also model experimental limitations by introducing a detection efficiency ε defined as the probability that a given locus present in a slice is detected. The expected fraction of nuclear profiles with an $\varepsilon < 1$, $N_{i,j}^{\varepsilon}$, is a linear combination of the $N_{i,j}$ values corresponding to $\varepsilon = 1$ (Beagrie et al., 2017). As an example, equation **Eq. 7** including a detection efficiency ε, becomes:

$$N_{1,1}^{\varepsilon} = \varepsilon^2 [N_{1,1} + (1-\varepsilon)\, 2N_{2,1} + (1-\varepsilon)^2 (2N_{3,1} + N_{2,2}) + (1-\varepsilon)^3\, 2N_{3,2} + (1-\varepsilon)^4\, 2N_{3,3}]$$

2.3 SLICE for multiplex-GAM datasets

In this section, we show how SLICE can be generalized for multiplex-GAM. In this new GAM protocol, multiple nuclear profiles are collected into a single sequencing tube. This leads to a significant reduction in reagent costs and labor time and a greater sensitivity (as we show in **Section 3**).

For any pair of loci, the previous equations for the single slices $N_{i,j}$ still hold true in multiplex-GAM, while the equations for the expected number of tubes with 0, 1 and 2 loci (M_0, M_1 and M_2) need to be modified to account for the number of nuclear profiles X_{NP} in each tube. By indicating with n the ploidy, we have:

$$\begin{aligned} M_0 &= (N_{0,0})^{XNP} \\ M_1 &= 2\,[(\textstyle\sum_{i=0}^{n} N_{i,0})^{XNP} - (N_{0,0})^{XNP}] \qquad \text{(Eq. 8)} \\ M_2 &= 1 - M_1 - M_0 \end{aligned}$$

Similar equations can be written for an arbitrary number of loci. For instance, in the case of triplets (A, B and C loci), we can first estimate the expected fraction of nuclear profiles, $N_{i,j,k}$ with a number i, j, k of loci A, B, C. From these, by analogous calculations, we can derive the expected fraction of tubes with 0,1,2 or 3 loci of the triplet (M_0, M_1, M_2 and M_3, respectively), in the general case of a number X_{NP} of nuclear profiles per each sequencing tube:

$$M_0 = (N_{0,0,0})^{XNP}$$

$$M_1 = [(\textstyle\sum_{i=0}^{n} N_{i,0,0})^{XNP} + (\sum_{j=0}^{n} N_{0,j,0})^{XNP} + (\sum_{k=0}^{n} N_{0,0,k})^{XNP} - 3(N_{0,0,0})^{XNP}]$$

$$M_2 = [(\textstyle\sum_{i,j=0}^{n} N_{i,j,0})^{XNP} + (\sum_{i,k=0}^{n} N_{i,0,k})^{XNP} + (\sum_{j,k=0}^{n} N_{0,j,k})^{XNP} - 2((\sum_{i=0}^{n} N_{i,0,0})^{XNP} + \sum_{j=0}^{n} N_{0,j,0})^{XNP} + \sum_{k=0}^{n} N_{0,0,k})^{XNP}) + 3N_{0,0,0}^{XNP}] \qquad \text{(Eq. 9)}$$

$$M_3 = 1 - M_2 - M_1 - M_0.$$

3. Using SLICE to optimize GAM experimental design

In this Section, we show how GAM sensitivity can be estimated by SLICE and how this can be used to optimize the GAM experimental parameters (e.g., the number of tubes to sequence, the slice thickness and the number of nuclear profiles per tube, etc.) for a given organism and cell type.

3.1GAM sensitivity

GAM sensitivity in the analysis of pairwise interactions can be defined as the probability to reject the null hypothesis H_0 that a pair of loci is non-interacting (Pi=0), when the alternative hypothesis H_1 is true, i.e. when the pair is interacting with a probability Pi>0.

As discussed in Beagrie et al., 2017, the interaction probability Pi can be estimated from GAM data by fitting the value of the co-segregation probability, i.e., $M_2/(M_1 + M_2)$, which, for loci at a given genomic distance, increases with increasing Pi. Beyond the expected value of the co-segregation ratio, its full probability distribution can be also estimated from SLICE (**Suppl. Notes Figure SN3a**).

By using the co-segregation ratio to estimate Pi, the GAM sensitivity Pr will depend on how distinct the probability distributions of the co-segregation ratio are for $Pi = 0$ and $Pi > 0$. Specifically, for any given combination of experimental parameters and interaction probability Pi, we can define Pr as the probability to obtain a co-segregation ratio $M_2/(M_2+M_1)$ greater than the 95th percentile of the distribution of co-segregation ratio at $Pi = 0$ (Beagrie et al., 2017, **Suppl. Notes Figure SN3a**).

Analogously, it is also possible to define GAM sensitivity to detect triplet interactions. Here, the co-segregation ratio is defined as $M_3/(M_1 + M_2 + M_3)$. Then, the sensitivity Pr is the probability to reject the null hypothesis $H_0 = \{Pi_3 = 0\}$ against the alternative hypothesis $H_1 = \{Pi_3 \neq 0\}$. In this case, Pr depends also on the pairwise interaction frequencies and we consider below two limit cases (Beagrie et al., 2017):

Case I: The three loci are able to interact only in a triplet (i.e., the pairwise interaction frequencies are all zero). In this scenario, the number of "false positives" (i.e., slices with all three loci present in a non-interacting state) is minimized.

Case II: Two of the loci making up the triplet are always engaged in a pairwise interaction (i.e., pairwise probability of interaction $Pi = 1$), and the third locus can bind to the pair and form a triplet. In this case, the number of "false positives" is maximum, as it is relatively easy to capture in a single slice all three loci while they do not form a triplet (it is as easy as capturing a pair of non-interacting loci).

As the Case I and Case II give, respectively, an overestimation and an underestimation of GAM sensitivity Pr, we expect that the true sensitivity falls between the values calculated in these two cases (Beagrie et al., 2017).

3.1Minimum number of tubes m*

GAM sensitivity Pr depends on several technical (e.g., detection efficiency, number of nuclear profiles per tube X_{NP}.) and biological (e.g., genomic distance, strength of interaction) factors. Importantly, Pr increases upon increase of sequenced tubes m (**Suppl. Notes Figure SN3b**). To provide a useful guide to the experimentalist who wishes to use GAM, we calculate the minimum number of tubes m^* required to reach a sensitivity greater or equal than 90% under different combinations of GAM parameters. The optimal choice of parameters minimizes the value of m^*.

Following this approach, we estimated m^* as a function of the number of nuclear profiles X_{NP} per tube and found that for currently used GAM parameters, $X_{NP} > 1$ (multiplex-GAM) typically gives higher sensitivity (**Extended Data Fig. 3c,e,f**; **Suppl. Notes Figure SN3c-d**). Moreover,

our results provide a guide to choose X_{NP} based on the values of the other parameters (eg, slice thickness) and the genomic distances (**Figure 1f,h**).

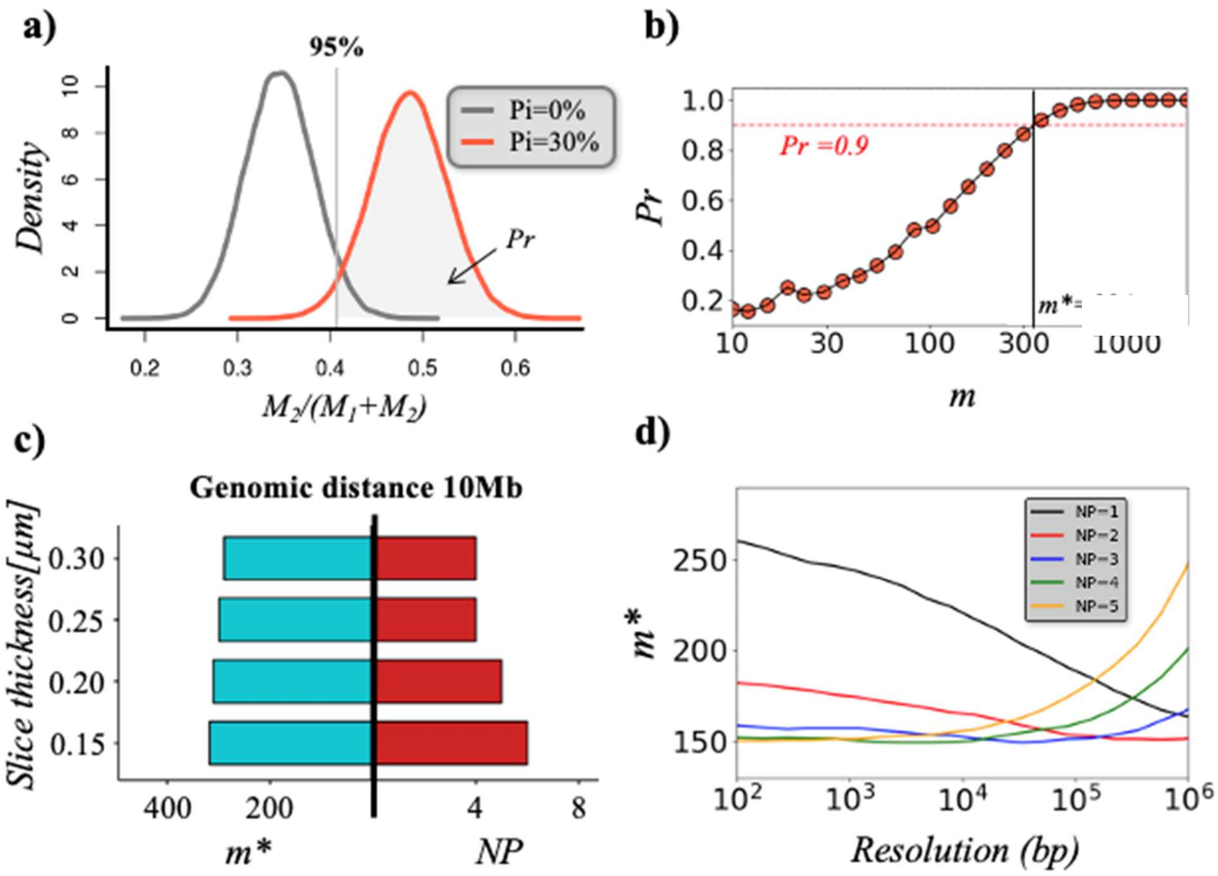

Figure SN3: Determining minimum number of tubes *m**.

a) The GAM sensitivity *Pr* (area under the red curve) is defined as the probability to reject the null hypothesis $H_0 = \{Pi = 0\}$, and is computed as the probability of obtaining a co-segregation ratio $M_2/(M_1+M_2)$ greater than the 95% percentile of co-segregation ratio distribution at $Pi = 0$. The distributions, shown here as example, are computed by setting $X_{NP} = 3$, genomic distance equal to *1Mb* and detection efficiency $\varepsilon=1$.
b) By studying the sensitivity *Pr* as a function of number of tubes, we can estimate the minimum number of tubes *m** that is necessary to detect a given pairwise interaction with a sensitivity greater or equal than a threshold (represented by a dashed line), that we fixed equal to 90% for our analyses. Here, we plot, as an example, the trend of *Pr* as a function of number of tubes, by setting $X_{NP} = 3$, genomic distance equal to *1Mb* and detection efficiency $\varepsilon=1$.
c) Optimal number of nuclear profiles per tube X_{NP} and the corresponding *m** for different values of the slice thickness *h*. Similarly to **Extended Data Fig. 3e**, we considered pairs of loci that are at a genomic distance of 10*Mb* and interaction probability *Pi* equal to 30%, but we set the experimental detection efficiency $\varepsilon=83\%$ as estimated from the original mESC GAM dataset (408x1*NP* at *30 kb* resolution; Beagrie et al., 2017). The optimal value for X_{NP} is consistent with the case $\varepsilon=1$.

d) m^* as a function of the resolution for different values of the number of nuclear profiles X_{NP}. Here, the detection efficiency ε is equal to *1*, the interaction probability Pi = 30%, and we considered pairs at large genomic distance (≥50Mb).

For triplet interactions, a good estimation of m^* is the average of the previously described Case I and Case II, (**Section 3.1, Suppl. Notes Figure SN4a**). As example, the heatmap in **Suppl. Notes Figure SN4b** shows m^* as a function of the triplet interaction probability Pi_3 and the number of nuclear profiles X_{NP}, for loci at 1Mb genomic distance. In this case, we find that when a small number of NPs are added per tube (i.e. $1 < X_{NP} < 10$), few tubes are sufficient to detect frequent triplet interactions (i.e. m^* is minimized).

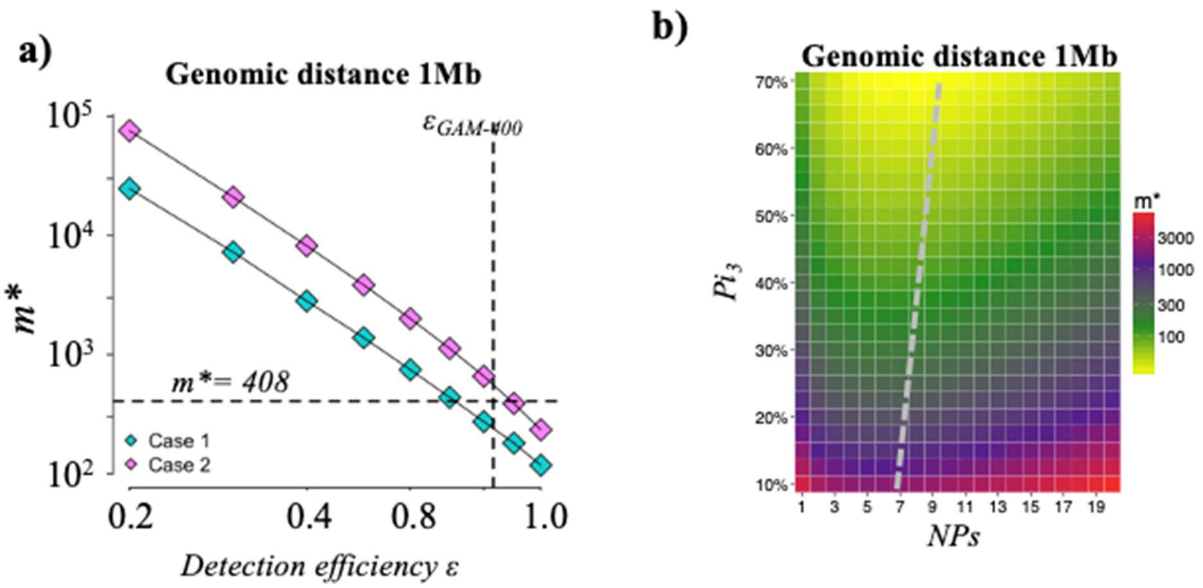

Figure SN4: Analysis of triplets.

a) m^* as a function of detection efficiency ε, where we fixed genomic distance equal to *1Mb* and interaction probability Pi = 50%. Here, we show the two limit cases, which give an overestimation and an underestimation of the sensitivity, respectively. The vertical dashed line marks the value of detection efficiency ε estimated from a previous GAM dataset (408x1*NP*) at 30 kb resolution, while the horizontal line marks the estimated number of tubes m^*.

b) Heatmap showing m^* as a function of the triplet interaction probability Pi_3 and the number of nuclear profiles X_{NP}. We considered a detection efficiency ε equal to 1 and triplets at genomic distance of *1Mb*. Note that the value of m^* plotted is the average of the m* values obtained in Case 1 and Case 2. The dashed grey line indicates the trend of the minimum m^*.

4. Analysis of Multiplex-GAM datasets

In **Section 2.3**, we showed how SLICE model can be extended to handle Multiplex-GAM datasets, in which multiple nuclear profiles are collected in each sequencing tube ($X_{NP}>1$). In this section, we test SLICE predictions on these new datasets and show that Multiplex-GAM reach a higher sensitivity. Next, we show that SLICE equations can handle combined GAM datasets with different nuclear profile per tube X_{NP}.

4.1SLICE predictions on multiplex GAM datasets ($X_{NP}>1$)

In **Section 2.3**, we showed how SLICE can be extended to consider datasets with $X_{NP}>1$. Through this improvement, we can estimate not only the minimal number of tubes m^* to achieve a given statistical power to reliably detect an interaction, but also the corresponding optimal value for X_{NP}, which represents an important parameter for the new Multiplex-GAM protocol (**Section 3**). Interestingly, for a constant number of tubes *m*, we find that GAM is more sensitive when multiple nuclear profiles *NPs* are in each tube (i.e. $X_{NP}>1$).

To quantitatively corroborate this prediction, we applied SLICE to two experimental datasets characterized by a different value of X_{NP}: the new Multiplex-GAM dataset, with m=249 tubes and $X_{NP}=3$ nuclear profiles per tube (briefly indicated by 249x3*NP*), and the 249x1*NP* dataset, that we generated i*n-silico* by randomly sampling a subset m=249 tubes from published data with $X_{NP}=1$ (408x1*NP*; Beagrie et al., 2017) (**Suppl. Notes Figure SN5b**). First, we compute the GAM sensitivity to detect pairwise interactions as a function of their genomic distances for both datasets (**Section 3.1**). As expected, we find a higher sensitivity for the 249x3*NP* dataset (blue bars) compared to the 249x1*NP* dataset (red bars), in the considered genomic distances (**Suppl. Notes Figure SN5a**). Here, we considered a detection efficiency ε equal to 1 and interaction probability equal to *Pi*=30%, but these results are robust to change of these parameters. Next, we run SLICE on both datasets and estimated the prominently interacting pairs, i.e. interactions with frequencies *Pi* greater than expected value *Pi* at a chosen threshold ($P \leq 0.05$, see Beagrie et al., 2017). By comparing the number of prominent pairwise interactions as a function of their genomic distance, we find that, as expected, more prominent interactions are detected in the 249x3*NP* dataset for all genomic distances (**Suppl. Notes Figure SN5c**), in agreement with the prediction.

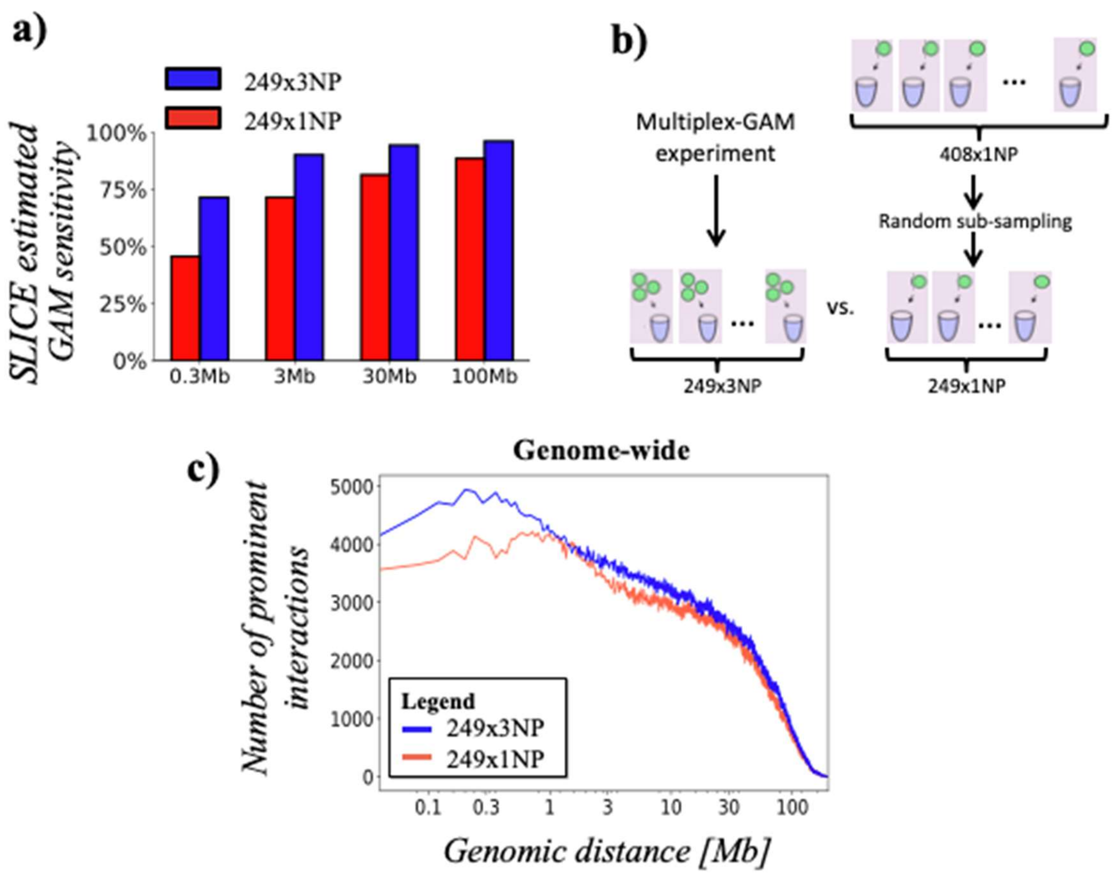

Figure SN5. Sensitivity of multiplex-GAM.

a) GAM sensitivity calculated by SLICE for two datasets with the same number of tubes (249) but different number of nuclear profiles per tube (X_{NP} = 1 and X_{NP} = 3, red and blue bars respectively), for some genomic distances. The sensitivity is predicted to be higher at all genomic distances for X_{NP} = 3. Here we fixed a detection efficiency equal to 1 and an interaction frequency Pi = 30%, but the results are robust to changes of these parameters.

b) We compared two GAM datasets with 249 tubes from mES cells: one generated with X_{NP}=3 (249x3NP) and another randomly sub-sampled from previously published data (Beagrie et al., 2017) with X_{NP}=1 (249x1NP).

c) The number of prominent interactions found by SLICE is plotted as a function of the genomic distance for the 249x3*NP* (blue line) and the 249x1NP GAM data (red line). As predicted by SLICE, in the 249x3*NP* dataset a larger number of interactions are systematically identified.

4.2SLICE predictions from combined datasets with variable X_{NP}

We next generalized SLICE to GAM datasets including tubes with a different number of nuclear profiles. To this aim, we used a mean-field approximation, consisting of introducing a number of nuclear profiles per tube (indicated by X_{MF}), that is the average of different number of nuclear profiles X_{NP}, weighted with corresponding number of tubes.

As a test, we applied SLICE to the GAM-1250 dataset in which the 481x1*NP* and the 249x3*NP* datasets are combined (also indicated by 481x1*NP* + 249x3*NP*). In this case, the estimated value of X_{MF} is:

$$X_{MF} = \frac{481x1+249x}{481+249} \approx 1.68$$

We run SLICE at 40 kb resolution and compared our results against the published GAM mESC-400 results at the same resolution. As expected, the detection efficiency ε estimated from the combined data (~86%) is comparable with the 408x1*NP* dataset at the same resolution (~93%) (Beagrie et al., 2017). Next, we compared the average probability u_0, estimated from the experimental datasets, as a function of genomic distance (**Suppl. Notes Figure SN6a**). This is an important quantity for numerically estimating interaction frequencies *Pi* and for assessing SLICE predictions (**Section 1-2** and (Beagrie et al., 2017)). We find that the trend of u_0 is very similar in both datasets and for all chromosomes, with Pearson correlation coefficients always around 98%.

Then, we looked at the distribution of differences of *Pi* values (**Suppl. Notes Figure SN6b**), in which, for sake of simplicity, we only show differences between values that are different from zero in both datasets. The mean value of the distribution is around zero and only low numerical differences can be found (max ~20%), proving that the two datasets have a similar distribution of *Pi*'s values. Moreover, we compared the most prominent interactions inferred from both datasets (**Section 3.1,** (Beagrie et al., 2017)). Specifically, for each chromosome we evaluated their overlap fraction (**Suppl. Notes Figure SN6c**) and computed the Pearson correlation coefficient between their common elements (**Suppl. Notes Figure SN6d**). Here, to take into account the effect of sparse matrices, we considered an element in common if the same element or at least one of its first neighbors was present.

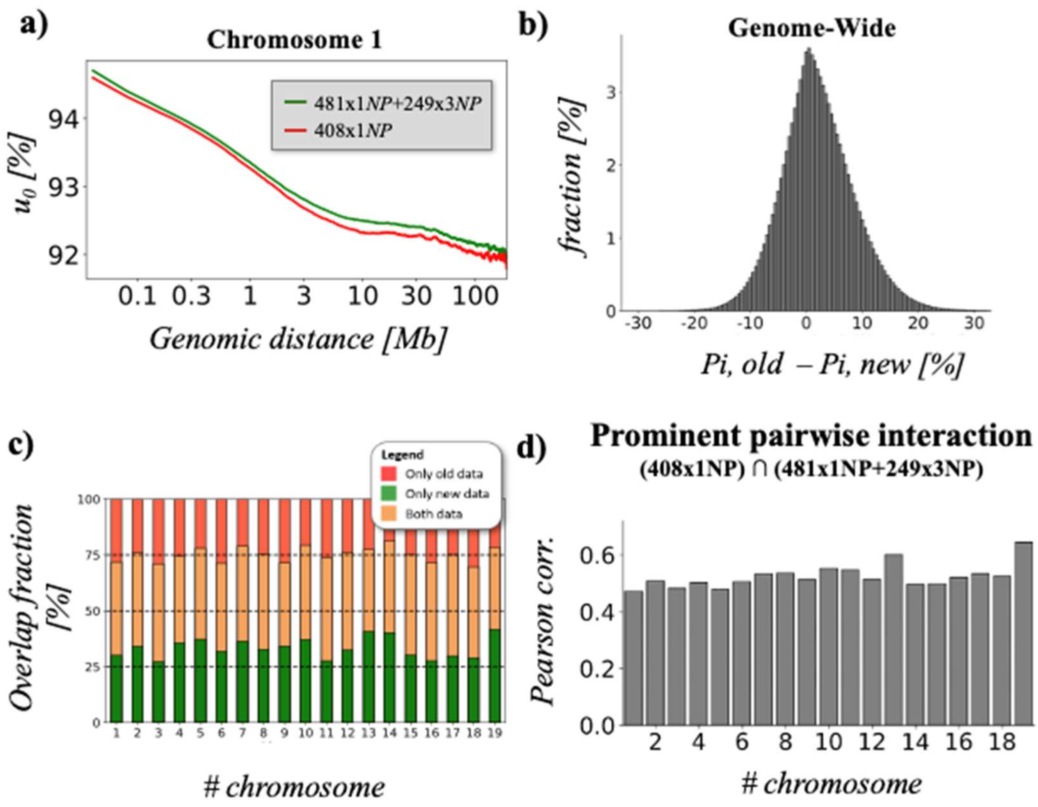

Figure SN6: SLICE results on Multiplex-GAM datasets.

a) The probability u_0 of not finding neither of two loci in a not interacting state, is an important fitting parameter for SLICE (Beagrie et al., 2017). Here, we compare the estimated trend of u_0 as a function of their genomic distance from previous 408x1*NP* and new combined dataset (481x1*NP*+249x3*NP*) for chromosome 1. The high Spearman correlation coefficient between the two trends (about 0.97), shows that the datasets provide very similar results.

b) Distribution of differences of *Pi* values inferred at 40 kb resolution from 408x1*NP* and combined 481x1*NP*+249x3*NP* data genome-wide. For sake of simplicity, here we show differences between *Pi* values that are different from zero in both datasets.

c) Overlap of most prominent contacts between *408x1NP* and combined *481x1NP+249x3NP* datasets, at *40 kb* resolution. The overlap is around 40% between prominent *Pi*'s in the two cases.

d) Pearson correlation coefficients between the prominent *Pi* matrices computed on *408x1NP* and *481x1NP+249x3NP* datasets. Here, we correlated only most prominent contacts that are present in both datasets.

Additional references

Beagrie, R.A., Scialdone, A., Schueler, M., Kraemer, D.C.A., Chotalia, M., Xie, S.Q., Barbieri, M., de Santiago, I., Lavitas, L., Branco, M.R., et al. (2017). Complex multi-enhancer contacts captured by genome architecture mapping. Nature *543*, 519–524.

Nagano, T., Lubling, Y., Várnai, C., Dudley, C., Leung, W., Baran, Y., Mandelson-Cohen, N., Wingett, S., Fraser, P., and Tanay, A. (2017). Cell cycle dynamics of chromosomal organisation at single-cell resolution. Nature.
