# Supplementary material for: Multiplex-GAM: genome-wide identification of chromatin contacts yields insights overlooked by Hi-C
Source: Nat Methods. 2023 Jun 19;20(7):1037–47. doi: 10.1038/s41592-023-01903-1 (PMC10333126; doi:10.1038/s41592-023-01903-1)
Supplement: Supplementary file 2 — Reporting Summary [file 41592_2023_1903_MOESM2_ESM.pdf]

nature research

Corresponding author(s): Ana Pombo, Mario Nicodemi & Lonnie R. Welch

Last updated by author(s): Feb 8, 2023

Reporting Summary

Nature Research wishes to improve the reproducibility of the work that we publish. This form provides structure for consistency and transparency in reporting. For further information on Nature Research policies, see our Editorial Policies and the Editorial Policy Checklist.

Statistics

For all statistical analyses, confirm that the following items are present in the figure legend, table legend, main text, or Methods section.

n/a	Confirmed	
☐	☒	The exact sample size (*n*) for each experimental group/condition, given as a discrete number and unit of measurement
☐	☒	A statement on whether measurements were taken from distinct samples or whether the same sample was measured repeatedly
☐	☒	The statistical test(s) used AND whether they are one- or two-sided<br>*Only common tests should be described solely by name; describe more complex techniques in the Methods section.*
☒	☐	A description of all covariates tested
☐	☒	A description of any assumptions or corrections, such as tests of normality and adjustment for multiple comparisons
☐	☒	A full description of the statistical parameters including central tendency (e.g. means) or other basic estimates (e.g. regression coefficient) AND variation (e.g. standard deviation) or associated estimates of uncertainty (e.g. confidence intervals)
☒	☐	For null hypothesis testing, the test statistic (e.g. *F*, *t*, *r*) with confidence intervals, effect sizes, degrees of freedom and *P* value noted<br>*Give P values as exact values whenever suitable.*
☒	☐	For Bayesian analysis, information on the choice of priors and Markov chain Monte Carlo settings
☒	☐	For hierarchical and complex designs, identification of the appropriate level for tests and full reporting of outcomes
☐	☒	Estimates of effect sizes (e.g. Cohen's *d*, Pearson's *r*), indicating how they were calculated

Our web collection on statistics for biologists contains articles on many of the points above.

Software and code

Policy information about availability of computer code

Data collection	No software was used for the collection of data in this study
Data analysis	GAM sequencing data was analysed using GAMtools v1.1.0, samtools v0.9, bedtools v2.27.1 and bowtie2 v2.1.0. Peaks in ChIP-seq data were called using BCP v1.1. Additional custom Python v3.7 scripts used for data analysis are available at https://github.com/pombo-lab/multiplex-gam-2022

For manuscripts utilizing custom algorithms or software that are central to the research but not yet described in published literature, software must be made available to editors and reviewers. We strongly encourage code deposition in a community repository (e.g. GitHub). See the Nature Research guidelines for submitting code & software for further information.

Data

Policy information about availability of data

All manuscripts must include a data availability statement. This statement should provide the following information, where applicable:

- Accession codes, unique identifiers, or web links for publicly available datasets
- A list of figures that have associated raw data
- A description of any restrictions on data availability

The original GAM sequencing data (Beagrie et al., 2017) are available from GEO (GSE64881). GAM sequencing data generated for this study are available as a separate accession (GSE166381). Hi-C data used are available from GEO (GSE35156). Other datasets (listed in Supplementary Table 4) are H3K9me3 (GSE18371), H4K20me3 (GSE12241), CTCF (GSE29184), Nanog (GSE11724), Oct4 (GSE11724), Sox2 (GSE11724), Tcf3 (GSE11724), Med12 (GSE22557), p300 (GSE29184), H3K27ac (GSE29184), GRO-seq (GSE27037), RNAPII-S7p (GSE94364), RNAPII-S5p (GSE94364), RNAPII-S2p (GSE34520), Total RNAPII (8GW16) (GSE94364), H3K36me3 (GSE11724), H3K79me2 (GSE11724), Jarid2 (GSE18776), Suz12 (GSE18776), Ezh2 (GSE18776), H3K27me3 (GSE94364), mESC HMM state calls (GSE17051)

Field-specific reporting

Please select the one below that is the best fit for your research. If you are not sure, read the appropriate sections before making your selection.

☒ Life sciences ☐ Behavioural & social sciences ☐ Ecological, evolutionary & environmental sciences

For a reference copy of the document with all sections, see nature.com/documents/nr-reporting-summary-flat.pdf

Life sciences study design

All studies must disclose on these points even when the disclosure is negative.

Sample size	The new GAM dataset was deemed sufficiently large because 80% of 40kb windows were detected at least 40 times.
Data exclusions	GAM datasets with <15% mapped reads were excluded from further analysis (Beagrie et al. 2017), as were all samples in experimental batches that were deemed to have failed (e.g. those stained with SybrGold, Extended Fig 4b). All GAM samples (including all excluded samples) are outlined in Supplementary Table 2 and all sequencing data is available under GEO accession GSE166381.
Replication	GAM datasets were derived from two biological replicates of mouse ES cells, independently grown at different times. Details of which samples belong to each replicate can be found in Supplementary Table 2. No significant difference was found between the two biological groups of samples.
Randomization	N/A (no experimental groups)
Blinding	N/A (no experimental groups)

Reporting for specific materials, systems and methods

We require information from authors about some types of materials, experimental systems and methods used in many studies. Here, indicate whether each material, system or method listed is relevant to your study. If you are not sure if a list item applies to your research, read the appropriate section before selecting a response.

Materials & experimental systems

n/a	Involved in the study	
☒	☐	Antibodies
☐	☒	Eukaryotic cell lines
☒	☐	Palaeontology and archaeology
☒	☐	Animals and other organisms
☒	☐	Human research participants
☒	☐	Clinical data
☒	☐	Dual use research of concern

Methods

n/a	Involved in the study	
☒	☐	ChIP-seq
☒	☐	Flow cytometry
☒	☐	MRI-based neuroimaging

Eukaryotic cell lines

Policy information about cell lines

Cell line source(s)	The mES cells used for this study were the 46C line, a Sox1–GFP derivative of E14tg2a and gift from D. Henrique.
Authentication	mES cell dentity was confirmed at the time of cryoblock creation by morphology and by confirming GFP expression after neural differentiation.
Mycoplasma contamination	mES cells were routinely tested for mycoplasma contamination and were found to be negative.
Commonly misidentified lines (See ICLAC register)	Neither 46C mES cells nor the parental line (E14tg2a) are listed on the ICLAC register
